# Nasal tophi

**DOI:** 10.1136/ard-2023-225246

**Published:** 2023-12-12

**Authors:** Xinxin Han, Yun Zhang

**Affiliations:** Department of Family Medicine & Division of General Internal Medicine, Peking Union Medical College Hospital, Chinese Academy of Medical Science & Peking Union Medical College, Beijing, People's Republic of China

**Keywords:** Gout, Arthritis, Vasculitis, Anti-Inflammatory Agents, Non-Steroidal

A 39-year-old man presented at our hospital due to recurrent swelling, redness and pain in his nasal area over the past 2 years. He felt that the dorsal hump of his nose had been progressively growing (figure 1)(A: frontal; B: half side; C: side). Physical examination revealed significant nasal swelling, erythema and tenderness on palpation, but no earlobe pain or swelling. His general condition was good with no underlying hypertension or diabetes mellitus. His only known comorbidity was gout, which was diagnosed 19 years ago. The patient was on urate-lowering therapy with febuxostat since the diagnosis. He intermittently tested his serum uric acid level, usually around 500 µmol/L. He had no family history of tumours or immune disease.

**Figure 1:**
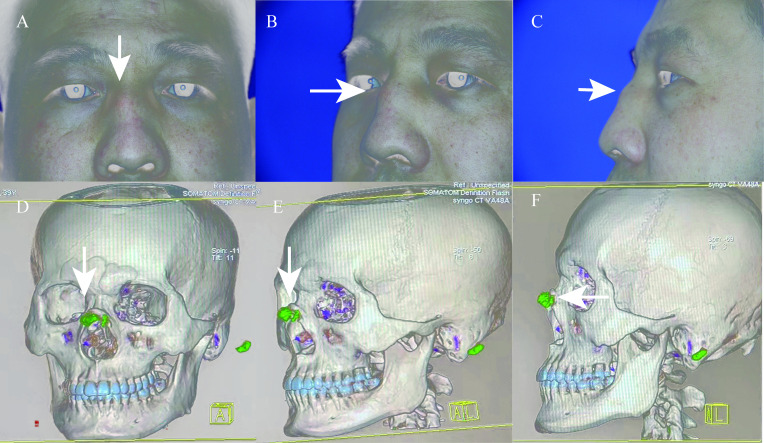
Nasal tophi (A: frontal; B: half side; C: side). Dual-energy CT scans identified monosodium urate crystal deposits within the nasal region (D: frontal; E: half side; F: side).

A comprehensive evaluation was undertaken. Laboratory analysis detected a blood uric acid level of 614 µmol/L. Dual-energy CT scans identified monosodium urate (MSU) crystal deposits within the nasal region (D: frontal; E: half side; F: side). Subsequent puncture confirmed the presence of MSU crystals under the microscope. No bacterial or fungal infection was found in the puncture. The patient exhibited gouty tophi in the nasal region. Following a 3-month course of anti-inflammatory treatment and urate-lowering therapy, the patient’s nasal pain improved, and the swelling gradually subsided. Nasal tophi, an exceedingly rare manifestation of gout, can mimic neoplasms or vasculitis and warrants careful differentiation.^1^ Recurrent inflammation of the nasal dorsum needs to be distinguished from nasal chondritis in relapsing polychondritis, which lacks specific diagnostic tests, making the diagnosis more challenging.^2^ When the diagnosis is difficult, puncture can be performed to exclude infections, tumours and other diseases. This case serves to enhance clinical awareness.

## References

[1] Sriranganathan MK , Vinik O , Bombardier C , et al . Interventions for Tophi in gout. Cochrane Database Syst Rev 2014:CD010069. 10.1002/14651858.CD010069.pub2 25330136

[2] Borgia F , Giuffrida R , Guarneri F , et al . Relapsing polychondritis: an updated review. Biomedicines 2018;6:84. 10.3390/biomedicines6030084 30072598 PMC6164217

